# Efforts to Develop Pfs25 Vaccines

**DOI:** 10.4269/ajtmh.21-1326

**Published:** 2022-07-11

**Authors:** David C. Kaslow

**Affiliations:** PATH Essential Medicines, PATH, Seattle, Washington

## Abstract

Acknowledging the fallibilities of recalling events from more than three decades ago, the recollection of Richard Carter’s impact on the identification and development of Pfs25, a major surface protein of *Plasmodium falciparum* zygotes and ookinetes, and target of malaria transmission-blocking vaccines, remains unassailable. In fondest memories of Richard Carter’s many contributions, herein retells some memorable events along the tortuous journey toward the development of Pfs25 vaccines.

## EFFORTS TO ISOLATE THE Pfs25 GENE

The early efforts to develop a Pfs25-based transmission-blocking vaccine built upon decades of observations in several species of animal malaria parasites, including the laboratory workhorse and avian malaria parasite *Plasmodium gallinaceum*. Following experiments in the 1950s by Huff et al.,^1^ Carter and Chen,^2^ and Gwadz^3^ independently published in 1976 that chickens immunized with *P. gallinaceum* sexual-stage parasites suppressed infectivity of subsequent blood infections to *Aedes* mosquitoes. In addition to identifying earlier expressed target antigens on *P. gallinaceum* gametocytes and gametes collected from infected chickens and *P. falciparum* gametocytes and gametes harvested from in vitro–cultured parasites, Carter’s laboratory identified and produced monoclonal antibodies (mAbs) to a major surface protein of *P. gallinaceum* zygotes (and ookinetes), subsequently named Pgs25.^4^ Meanwhile, an analogous major surface protein of *P. falciparum* zygotes (and ookinetes), Pfs25, and a series of mAbs thereto, some of which interfered with mosquito infectivity and others that did not, were reported by the Meuwissen laboratory in Nijmegen, The Netherlands.^5^ Although in vitro–cultured gametocytes and gametes synthesized Pfs25 at detectable levels, Pfs25 only became a major surface protein when subsequent zygotes transformed to ookinetes.^5^ Large-scale passage of *P. gallinaceum* in chickens and in vitro culture of *P. falciparum* gametocytes, coupled with the development in the Carter laboratory of mAb 1C7 by Quakyi et al. (unpublished) to purify Pfs25,^6^ set the stage for simultaneous and parallel efforts to clone the genes for Pfs25 and Pgs25.

Initial attempts to clone the Pfs25 gene by immuno-screening procaryotic expression libraries failed, likely because the highly reducing environment within *Escherichia coli* did not recreate the disulfide bonds required for recognition by the conformation-dependent mAbs and, as subsequently observed, the inherent toxicity of recombinant Pfs25 expression in *E. coli*. In 1986, purifying enough protein to determine an amino acid sequence from which to the synthesize degenerate oligonucleotides for screening genomic or complementary DNA libraries seemed the next best alternative. The same approach was taken simultaneously for both the chicken and human malaria parasite models—namely, immunoaffinity chromatography followed by sodium dodecyl-sulfate–polyacrylamide gel electrophoresis (SDS-PAGE) to purify tens of micrograms of Pgs25 from 50 to 100 *P. gallinaceum*–infected chickens, or tens of micrograms of Pfs25 from 3 months of in vitro–cultured *P. falciparum* gametocytes. As has been the subsequent journey for Pfs25, dead ends occurred frequently.

The first protein micro-sequenced was a contaminant: the light chain of immunoglobin, which had leached from the immunoaffinity chromatography and co-migrated at approximately 25 kDa. To distinguish between the mAb used to purify parasite proteins and the proteins of interest, bulk parasite extracts were spiked with metabolically ^35^S-labeled parasite proteins as a tracer. From another 50 to 100 chickens, and 3 months of in vitro culture, highly purified parasite-produced protein was eluted from SDS-PAGE gel slices and micro-sequenced by automated Edman degradation. Unfortunately, the NH_2_-terminus of the mature proteins were blocked, preventing NH_2_-terminal amino acid sequencing. Another round of purification, to produce tryptic peptides for sequencing internal peptides fragment, revealed resistance of the disulfide-rich protein to trypsin digestion, absent denaturation and alkylation. Finally, one of three tryptic peptide fragment peaks generated from reduced and alkylated Pfs25 provided an amino acid sequence for constructing degenerate synthetic oligonucleotides. The first round of screening hundreds of thousands of bacteriophage plaques and plasmid colonies yielded no signal. Neither did Southern or Northern blots. At that point, it became clear that the nucleotide sequence was not present in the *P. falciparum* genome. Re-analysis of the original micro-sequence data revealed an error in reading one of the amino acid residues. The corrected degenerate synthetic oligonucleotides were found to be too degenerate for screening genomic libraries, but hybridized specifically to an abundant transcript in *P. falciparum* gametocytes and zygotes by Northern blot analysis. After reducing the degeneracy of the proline codon in the center of the oligonucleotide probe, the Pfs25 gene was isolated and its sequence determined.^6^

A search of protein databases with the deduced amino acid sequence from Pfs25 revealed homology to a diverse set of proteins that all contained epidermal growth factor-like domains. Between the putative NH_2_-terminal secretory signal sequence and a short hydrophobic region at the COOH-terminus that presumably signaled transfer to a glycosylphosphatidylinositol membrane anchor, Pfs25 had four such domains (the first of which appears truncated). Epidermal growth factor-like domains consist of six cysteine residues that form three disulfide bonds, consistent with the observation that all the transmission-blocking mAbs to Pfs25 available at the time recognized disulfide bond-dependent epitopes. This secondary structure provided a significant challenge in producing recombinant Pfs25 vaccine candidates capable of inducing potent polyclonal transmission-blocking antibodies.

## EFFORTS TO PRODUCE EFFECTIVE Pfs25 IMMUNOGENS

With gene in hand, creating recombinant Pfs25 immunogens also took two simultaneous journeys,^7^ both of which continue today: 1) subunit protein expression and 2) transgene delivery by viral vectors and nucleic acid vaccines. Although efforts to express full-length Pfs25 in off-the-shelf *E. coli* expression systems in the late 1980s provided little to no soluble or secreted protein, abundant amounts of fusion protein comprised of truncated recombinant Pfs25, without the putative NH_2_-terminal signal sequence and COOH-terminus glycosylphosphatidylinositol anchor sequence, could be recovered as inclusion bodies. The solubilized Pfs25 failed to induce potent transmission-blocking antibodies in mice, even when formulated with potent adjuvants, likely because of a failure to recreate the disulfide bonds in a recombinant protein that has 22 cysteines.^7^ Subsequent efforts turned to *Saccharomyces cerevisiae* expression systems, first with a team lead by Dr. Phillip Barr at Chiron Corporation,^8^ and subsequently in collaboration with Dr. Joseph Shiloach and Dr. Anthony Stowers at US National Institutes of Health,^9^^,^^10^ and, for clinical investigational product, Dr. Ginny Price at Immunex.^7^ More recently, Pfs25 expressed in *Pichia pastoris*,^11^ wheat germ extract,^12^ baculovirus,^13^ plants,^14^ algae,^15^ and bacteria (codon harmonized and refolded)^16^ have been evaluated in animal immunogenicity studies, with the plant-based Pfs25 virus-like particle (developed by Fraunhofer USA Center for Molecular Biotechnology) advancing to human clinical trials.^17^ Beyond soluble Pfs25 monomeric vaccine candidates, which have proved to be poor immunogens, animal immunogenicity studies of Pfs25 protein conjugates, including to carrier protein,^18^ outer-membrane protein complex,^19^ and as nanoparticles,^20^ have also led to human clinical trials.^21^

Although attempting unsuccessfully to make *E. coli*--produced recombinant protein subunit vaccine candidates that either bound transmission-blocking antibodies or elicited potent transmission-blocking antibodies in laboratory animal models,^22^ a collaboration with Dr. Stuart Isaacs in Dr. Bernard Moss’s laboratory yielded a recombinant Western Reserve strain of vaccinia virus, vSIDK, that encoded full-length Pfs25. A series of conformational-dependent transmission-blocking mAbs recognized the surface of live vSIDK-infected mammalian cells expressing Pfs25. Despite eliciting antibodies to Pfs25, mice vaccinated once with vSIDK failed to elicit transmission-blocking antibodies. However, inoculating mice thrice with vSIDK elicited polyclonal antibodies with transmission-blocking potency superior to mAbs.^23^ Because the safety profile of the virulent Western Reserve strain was not suitable for use as a transmission-blocking vaccine, particularly in populations with significant numbers of immunocompromised individuals (e.g., persons living with HIV), the viral vector effort turned to a large collaborative public–private partnership to develop an attenuated strain of recombinant vaccinia virus (NYVAC-Pf7) that expressed Pfs25, among other target antigens from the liver and blood stages of the *falciparum* parasite.^24^ That effort led to the first human clinical trial of Pfs25.^25^ More recently, several gene-based delivery systems have been tested as vehicles for eliciting Pfs25 transmission-blocking antibodies, including DNA,^26^ modified vaccinia Ankara,^27^ chimpanzee adenovirus,^27^ human adenovirus,^27^^,^^28^ and adeno-associated virus,^29^ with a heterologous vaccinia and adenovirus series recently advancing to first-in-human studies.^30^

## EFFORTS TO EVALUATE Pfs25 VACCINE CANDIDATES IN HUMANS

By the mid-1990s, three clinical trials of transmission-blocking vaccines had been conducted: one with a recombinant subunit protein, one with a viral vector, and one combining both modalities. The first Pfs25 phase I trial evaluated the highly attenuated vaccinia virus NYVAC-Pf7, which encoded seven malaria parasite antigens, one of which was full-length Pfs25.^25^ Data from preclinical studies in experimental laboratory animals indicated that Pfs25 was immunogenic,^24^ eliciting transmission-reducing activity (unpublished data)^7^; however, neither animals nor humans elicited humoral responses with complete transmission-blocking activity.

In 1994, the second human trial evaluated yeast-produced Pfs25 adsorbed to Alhydrogel^®^ (Biosector A/S [now Croda], Frederikssund, Denmark) in healthy adults administered three monthly doses of TBV25H/alum. All seven subjects that received three doses seroconverted by ELISA, but none developed transmission-blocking activity by a standard membrane-feeding assay. One of seven volunteers who received a third dose of TBV25H/alum experienced pruritic swelling within 30 minutes, initially at the site of the third dose of the vaccine and within several hours at the site of the previous second dose (contralateral deltoid). Erythema distal to the third injection site at 48 hours resolved with oral antihistamine and topical corticosteroid cream. In addition to five others who received a third dose of TBV25H/alum, the subject had a transient increase in eosinophils to the high end of the normal range.

To explore the hypothesis that free antigen dissociated from the alum resulted in the atypical contralateral hypersensitivity reaction, mice that had received two high doses of TBV25H/alum intraperitoneally were administered either a third high dose of TBV25H/alum: the supernatant from centrifuged high-dose TBV25H/alum or the resuspended pellet from centrifuged high-dose TBV25H/alum. Mice that received a third dose of TBV25H/alum or the supernatant thereof developed an acute reaction characterized by temporary lordosis (hunching), rough hair coat, and rarely death (3 in 100 mice). Mice that received the resuspended pellet did not demonstrate a similar reaction. By reducing the pH of the phosphate buffer used during final formulation, the percentage of TBV25H bound to alum increased without compromising stability.^31^ This formulation of TBV25H advanced to the heterologous regimen with NYVAC-Pf7, in which nine subjects previously vaccinated with NYVAC-Pf7 and four unvaccinated control subjects received a single dose of TBV25H/alum. Pre-dose and 14 days post-dose serum samples demonstrated for the first time the biological feasibility of eliciting transmission-blocking antibodies in humans with a Pfs25-based vaccine.^31^

## ATTRACTION AND ATTRITION OF Pfs25 VACCINES

The initial attraction of Pfs25 included its validation as a target of transmission-blocking immunity induced by vaccination with zygotes, modest size for recombinant protein expression, minimal genetic diversity,^32^ absence of genetic restriction in immunogenicity studies with H-2 congenic mice,^33^ and apparent muted translation despite abundant transcription while gametocytes circulate in the human host (i.e., lack of immune selection as a consequence of expression while in the human host).^5^^,^^6^ The now 33-year journey, since the isolation of the Pfs25 gene, to a well-tolerated and efficacious Pfs25 vaccine for use in humans has been tortuous and is far from over, with attrition in human safety and immunogenicity studies of too many candidates to recount here. The biggest hurdle remains one that Richard Carter recognized long ago: the lack of an immune response in humans.^34^ Recent studies suggest an affinity maturation pathway of as little as seven amino acid changes from an inferred germline B-cell precursor to a highly potent transmission-blocking antibody in humans,^35^ whereas another study cautions that Pfs25 may lack dominant human major histocompatibility complex class II–restricted T-cell epitopes to elicit such antibodies.^36^ Together the findings provide potential biomarkers and vaccine design changes to inform another generation of Pfs25-based vaccine candidates that may achieve Richard Carter’s vision of highly efficacious and durable malaria parasite transmission-blocking vaccines.

## References

[1] Huff CG Marchbank DF Shiroishi T , 1958. Changes in infectiousness of malarial gametocytes: II. Analysis of the possible causative factors. Exp Parasitol 7: 399–417. 13562104 10.1016/0014-4894(58)90036-5

[2] Carter R Chen DH , 1976. Malaria transmission blocked by immunisation with gametes of the malaria parasite. Nature 263: 57–60. 986561 10.1038/263057a0

[3] Gwadz RW , 1976. Malaria: successful immunization against the sexual stages of *Plasmodium gallinaceum* . Science 193: 1150–1151. 959832 10.1126/science.959832

[4] Grotendorst CA Kumar N Carter R Kaushal DC , 1984. A surface protein expressed during the transformation of zygotes of *Plasmodium gallinaceum* is a target of transmission-blocking antibodies. Infect Immun 45: 775–777. 6540756 10.1128/iai.45.3.775-777.1984 PMC263365

[5] Vermeulen AN Ponnudurai T Beckers PJ Verhave JP Smits MA Meuwissen JH , 1985. Sequential expression of antigens on sexual stages of *Plasmodium falciparum* accessible to transmission-blocking antibodies in the mosquito. J Exp Med 162: 1460–1476. 2865324 10.1084/jem.162.5.1460 PMC2187939

[6] Kaslow DC Quakyi IA Syin C Raum MG Keister DB Coligan JE McCutchan TF Miller LH , 1988. A vaccine candidate from the sexual stage of human malaria that contains EGF-like domains. Nature 333: 74–76. 3283563 10.1038/333074a0

[7] Paoletti LC McInnes PM , eds., 1999. Vaccines, from Concept to Clinic: A Guide to the Development and Clinical Testing of Vaccines for Human Use. Boca Raton, FL: CRC Press.

[8] Barr PJ Green KM Gibson HL Bathurst IC Quakyi IA Kaslow DC , 1991. Recombinant Pfs25 protein of *Plasmodium falciparum* elicits malaria transmission-blocking immunity in experimental animals. J Exp Med 174: 1203–1208. 1940798 10.1084/jem.174.5.1203 PMC2118997

[9] Kaslow DC Shiloach J , 1994. Production, purification and immunogenicity of a malaria transmission-blocking vaccine candidate: TBV25H expressed in yeast and purified using nickel-NTA agarose. Biotechnology (N Y) 12: 494–499. 7764708 10.1038/nbt0594-494

[10] Stowers AW Keister DB Muratova O Kaslow DC , 2000. A region of *Plasmodium falciparum* antigen Pfs25 that is the target of highly potent transmission-blocking antibodies. Infect Immun 68: 5530–5538. 10992450 10.1128/iai.68.10.5530-5538.2000 PMC101502

[11] Zou L Miles AP Wang J Stowers AW , 2003. Expression of malaria transmission-blocking vaccine antigen Pfs25 in *Pichia pastoris* for use in human clinical trials. Vaccine 21: 1650–1657. 12639486 10.1016/s0264-410x(02)00701-6

[12] Tsuboi T 2008. Wheat germ cell-free system-based production of malaria proteins for discovery of novel vaccine candidates. Infect Immun 76: 1702–1708. 18268027 10.1128/IAI.01539-07 PMC2292889

[13] Mlambo G Kumar N Yoshida S , 2010. Functional immunogenicity of baculovirus expressing Pfs25, a human malaria transmission-blocking vaccine candidate antigen. Vaccine 28: 7025–7029. 20709008 10.1016/j.vaccine.2010.08.022

[14] Farrance CE 2011. Antibodies to plant-produced *Plasmodium falciparum* sexual stage protein Pfs25 exhibit transmission blocking activity. Hum Vaccin 7: 191–198. 10.4161/hv.7.0.14588 21266847

[15] Gregory JA Li F Tomosada LM Cox CJ Topol AB Vinetz JM Mayfield S , 2012. Algae-produced Pfs25 elicits antibodies that inhibit malaria transmission. PLoS One 7: e37179. 22615931 10.1371/journal.pone.0037179 PMC3353897

[16] Kumar R Angov E Kumar N , 2014. Potent malaria transmission-blocking antibody responses elicited by *Plasmodium falciparum* Pfs25 expressed in *Escherichia coli* after successful protein refolding. Infect Immun 82: 1453–1459. 24421036 10.1128/IAI.01438-13 PMC3993404

[17] Chichester JA 2018. Safety and immunogenicity of a plant-produced Pfs25 virus-like particle as a transmission blocking vaccine against malaria: a phase 1 dose-escalation study in healthy adults. Vaccine 36: 5865–5871. 30126674 10.1016/j.vaccine.2018.08.033 PMC6143384

[18] Kubler-Kielb J Majadly F Wu Y Narum DL Guo C Miller LH Shiloach J Robbins JB Schneerson R , 2007. Long-lasting and transmission-blocking activity of antibodies to *Plasmodium falciparum* elicited in mice by protein conjugates of Pfs25. Proc Natl Acad Sci USA 104: 293–298. 17190797 10.1073/pnas.0609885104 PMC1765452

[19] Wu Y 2006. Sustained high-titer antibody responses induced by conjugating a malarial vaccine candidate to outer-membrane protein complex. Proc Natl Acad Sci USA 103: 18243–18248. 17110440 10.1073/pnas.0608545103 PMC1636993

[20] Shimp RL 2013. Development of a Pfs25-EPA malaria transmission blocking vaccine as a chemically conjugated nanoparticle. Vaccine 31: 2954–2962. 23623858 10.1016/j.vaccine.2013.04.034 PMC3683851

[21] Talaat KR 2016. Safety and immunogenicity of Pfs25-EPA/Alhydrogel^®^, a transmission blocking vaccine against *Plasmodium falciparum*: an open label study in malaria naïve adults. PLoS One 11: e0163144. 27749907 10.1371/journal.pone.0163144 PMC5066979

[22] Kaslow DC Bathurst IC Lensen T Ponnudurai T Barr PJ Keister DB , 1994. *Saccharomyces cerevisiae* recombinant Pfs25 adsorbed to alum elicits antibodies that block transmission of *Plasmodium falciparum.* Infect Immun 62: 5576–5580. 7960139 10.1128/iai.62.12.5576-5580.1994 PMC303304

[23] Kaslow DC Isaacs SN Quakyi IA Gwadz RW Moss B Keister DB , 1991. Induction of *Plasmodium falciparum* transmission-blocking antibodies by recombinant vaccinia virus. Science 252: 1310–1313. 1925544 10.1126/science.1925544

[24] Tine JA 1996. NYVAC-Pf7: a poxvirus-vectored, multiantigen, multistage vaccine candidate for *Plasmodium falciparum* malaria. Infect Immun 64: 3833–3844. 8751936 10.1128/iai.64.9.3833-3844.1996 PMC174300

[25] Ockenhouse CF 1998. Phase I/IIa safety, immunogenicity, and efficacy trial of NYVAC-Pf7, a pox-vectored, multiantigen, multistage vaccine candidate for *Plasmodium falciparum* malaria. J Infect Dis 177: 1664–1673. 9607847 10.1086/515331

[26] Lobo CA Dhar R Kumar N , 1999. Immunization of mice with DNA-based Pfs25 elicits potent malaria transmission-blocking antibodies. Infect Immun 67: 1688–1693. 10085005 10.1128/iai.67.4.1688-1693.1999 PMC96515

[27] Goodman AL Blagborough AM Biswas S Wu Y Hill AV Sinden RE Draper SJ , 2011. A viral vectored prime-boost immunization regime targeting the malaria Pfs25 antigen induces transmission-blocking activity. PLoS One 6: e29428. 22216279 10.1371/journal.pone.0029428 PMC3247263

[28] McGuire KA Miura K Wiethoff CM Williamson KC , 2017. New adenovirus-based vaccine vectors targeting Pfs25 elicit antibodies that inhibit *Plasmodium falciparum* transmission. Malar J 16: 254. 28619071 10.1186/s12936-017-1896-7 PMC5471885

[29] Yusuf Y 2019. Adeno-associated virus as an effective malaria booster vaccine following adenovirus priming. Front Immunol 10: 730. 31024558 10.3389/fimmu.2019.00730 PMC6460511

[30] de Graaf H 2021. Safety and immunogenicity of ChAd63/MVA Pfs25-IMX313 in a phase I first-in-human trial. Front Immunol 12: 694759. 34335606 10.3389/fimmu.2021.694759 PMC8318801

[31] Kaslow DC , 2002. Transmission-blocking vaccines. Malaria Immunol 80: 287–307. 10.1159/000058850 12058646

[32] Kaslow DC Quakyi IA Keister DB , 1989. Minimal variation in a vaccine candidate from the sexual stage of *Plasmodium falciparum.* Mol Biochem Parasitol 32: 101–103. 2643034 10.1016/0166-6851(89)90134-5

[33] Good MF Miller LH Kumar S Quakyi IA Keister D Adams JH Moss B Berzofsky JA Carter R , 1988. Limited immunological recognition of critical malaria vaccine candidate antigens. Science 242: 574–577. 2902690 10.1126/science.2902690

[34] Carter R Graves PM Quakyi IA Good MF , 1989. Restricted or absent immune responses in human populations to *Plasmodium falciparum* gamete antigens that are targets of malaria transmission-blocking antibodies. J Exp Med 169: 135–147. 2642527 10.1084/jem.169.1.135 PMC2189199

[35] McLeod B 2019. Potent antibody lineage against malaria transmission elicited by human vaccination with Pfs25. Nat Commun 10: 4328. 31551421 10.1038/s41467-019-11980-6 PMC6760140

[36] Zaric M 2021. Poor CD4+ T cell immunogenicity limits humoral immunity to *P. falciparum* transmission-blocking candidate Pfs25 in humans. Front Immunol 12: 732667. 34659219 10.3389/fimmu.2021.732667 PMC8515144

